# Increased Added Sugar Consumption Is Common in Parkinson's Disease

**DOI:** 10.3389/fnut.2021.628845

**Published:** 2021-05-07

**Authors:** Natalie C. Palavra, Michal Lubomski, Victoria M. Flood, Ryan L. Davis, Carolyn M. Sue

**Affiliations:** ^1^Department of Neurology, Royal North Shore Hospital, Sydney, NSW, Australia; ^2^Department of Neurogenetics, Kolling Institute, University of Sydney and Northern Sydney Local Health District, Sydney, NSW, Australia; ^3^School of Medicine, The University of Notre Dame Australia, Sydney, NSW, Australia; ^4^School of Health Sciences, Faculty of Medicine and Health, University of Sydney, Camperdown, NSW, Australia; ^5^Allied Health Research Unit, Westmead Hospital, Western Sydney Local Health District, Sydney, NSW, Australia

**Keywords:** Parkinson's disease, diet, nutrition, carbohydrates, sugars

## Abstract

**Objectives:** There is limited information about the dietary habits of patients with Parkinson's Disease (PD), or associations of diet with clinical PD features. We report on nutritional intake in an Australian PD cohort.

**Methods:** 103 PD patients and 81 healthy controls (HCs) completed a validated, semi-quantitative food frequency questionnaire. Food and nutrient intake was quantified, with consideration of micronutrients and macronutrients (energy, protein, carbohydrate, fat, fibre, and added sugar). Participants also completed PD-validated non-motor symptom questionnaires to determine any relationships between dietary intake and clinical disease features.

**Results:** Mean daily energy intake did not differ considerably between PD patients and HCs (11,131 kJ/day vs. 10,188 kJ/day, *p* = 0.241). However, PD patients reported greater total carbohydrate intake (279 g/day vs. 232 g/day, *p* = 0.034). This was largely attributable to increased daily sugar intake (153 g/day vs. 119 g/day, *p* = 0.003) and in particular free sugars (61 g/day vs. 41 g/day, *p* = 0.001). PD patients who (1) experienced chronic pain, (2) were depressed, or (3) reported an impulse control disorder, consumed more total sugars than HCs (all *p* < 0.05). Increased sugar consumption was associated with an increase in non-motor symptoms, including poorer quality of life, increased constipation severity and greater daily levodopa dose requirement.

**Conclusions:** We provide clinically important insights into the dietary habits of PD patients that may inform simple dietary modifications that could alleviate disease symptoms and severity. The results of this study support clinician led promotion of healthy eating and careful management of patient nutrition as part of routine care.

## Introduction

Parkinson's Disease (PD) is the second most common neurodegenerative disease and is associated with significant morbidity (1). It is characterised by the loss of dopaminergic neurons in the substantia nigra pars compacta, and a deficiency of dopamine in the striatum and other basal ganglia structures. A growing body of evidence suggests that nutrition may play an important role in PD (2).

PD patients are more frequently underweight (3, 4), have a higher risk of malnutrition (5) and tend to have a lower body mass index (BMI) (6) that inversely associates with disease duration, disease severity and levodopa-related motor complications (7). Furthermore, it has been suggested that lower dietary intake of poly-unsaturated fatty acids, vitamin A, vitamin E, vitamin B12, vitamin D and folic acid are associated with an increased risk of developing PD (8, 9), although this remains controversial. Nevertheless, throughout the disease course, weight gain and loss may fluctuate, being influenced by both changes in food intake and energy expenditure (10). Interestingly, PD patients are also purported to display a preference for sweet foods, such as cakes (11), chocolate (12), ice cream (13), milk puddings and custards (14), consistent with an increased consumption of carbohydrates (7, 15, 16).

Emerging research suggests that the complex bidirectional communication between the gut and brain is influenced by dietary patterns and may contribute to the development and progression of PD (17, 18), as well as levodopa metabolism (19). Therefore, the predominance of gastrointestinal dysfunction in PD may further influence the diet of PD patients and vice versa. For example, PD patients are three-times more likely to experience constipation than control subjects and they reported increased occurrence and severity of indigestion, nausea, excessive fullness and bloating (20), which negatively impact on PD health-related quality of life (QoL) (21). Additionally, constipation associates with higher levodopa requirements (7), likely due to gastroparesis and impaired intestinal motility, which hinders drug absorption.

Despite ethnic variability in dietary habits (22), general improvement in nutritional condition has been shown to improve PD patient QoL (23). Furthermore, adherence to a healthy diet may reduce the occurrence of non-motor symptoms that often precede PD diagnosis (24) and may lead to optimisation of levodopa therapy to minimise disease-associated complications (7). Due to limited information about the dietary habits of PD patients in Australia, we aimed to characterise the nutritional intake of an Australian PD cohort, and investigate potential associations between diet and clinical disease features.

## Materials and Methods

### Study Settings and Subjects

Subjects were recruited from the movement disorder and neurology clinics at Royal North Shore Hospital, Sydney, Australia, between 2018 and 2019, as described previously (20). Inclusion criteria were; >18 years of age, a clinical diagnosis of idiopathic PD according to the UK Parkinson's Disease Society Brain Bank Diagnostic Criteria (25), regardless of disease duration, and being managed by a specialist neurologist. The healthy control (HC) inclusion criteria were; >18 years of age, exhibiting no clinical indication of PD, and were a spouse, sibling or child of a respective PD patient with similar dietary habits. Exclusion criteria included secondary Parkinsonism, tube feeding, medical or surgical disorders preventing completion of questionnaires and significant cognitive impairment demonstrated by incapacity to provide consent. Ethical approval was granted by the Northern Sydney Local Health District Human Research Ethics Committee and the North Shore Private Hospital Ethics Committee, HREC/18/HAWKE/109, NSPHEC 2018-LNR-009, respectively.

### Data Collection

Dietary and lifestyle data were collected for all participants through a 145-item, semi-quantitative food frequency questionnaire (FFQ), modified for Australian diet and vernacular from an early Willett FFQ (26, 27) and originally developed and validated as part of the Blue Mountains Eye Study (28, 29). The FFQ was later updated to reflect new foods commonly available in the Australian food supply (30). A nine-category frequency scale was used to indicate the usual frequency of consumption of food items during the past year, and included portion size estimates. Nutrient content of food items was calculated using the Australian Food Composition Database (31) multiplied by the frequency and portion size, using a purpose-built Microsoft Access program. Nutrient analysis included calculations for energy, protein, carbohydrate, sugars, fats and fibre. “Added sugars” include sucrose, fructose, dextrose, lactose and sugar syrups (such as glucose syrup), which are added during the manufacture of foods or by the consumer in the preparation of food and beverages (32). “Free sugars” extends the definition of added sugars to include sugars naturally present in honey, fruit juice and fruit juice concentrates (33). Total average daily consumption of macronutrients and micronutrients were calculated. Macronutrients were also calculated as a percentage of total energy intake and micronutrients per 1,000 kJ (34).

Demographic data was collected on patient age, gender, ethnicity, marital status, socioeconomic status and medical history, including co-morbidities, medication use, alcohol consumption and smoking history. Physical activity was assessed using the International Physical Activity Questionnaire (35). The Leeds Dyspepsia Questionnaire (36) assessed upper gastrointestinal symptoms and the Rome-IV criteria (37) and the Cleveland Constipation Score (38) were used to determine constipation severity and gut motility. QoL was evaluated using the PDQ-39 (39), mood was assessed by the Beck Depression Inventory (40), cognitive function was gauged by the Montreal Cognitive Assessment (MoCA) (41) and non-motor symptoms were assessed by the Non-Motor Symptoms Scale (NMSS) (42). Quantitative and qualitative motor severity assessment was evaluated with the Movement Disorder Society—Unified Parkinson's Disease Rating Scale—Part III (MDS-UPDRS III) and the Modified Hoehn & Yahr scale (43). Medications were compared following standard methods for calculating daily levodopa equivalent dose (LED) (44), whilst chronic pain severity was assessed by the Visual Analogue Scale (45).

### Statistical Analysis

Normal distribution of all data was confirmed using the Shapiro-Wilk test. Independent *t*-tests were used to analyse differences between groups for continuous variables. Chi-squared (χ^2^) tests were used to compare differences between categorical variables. Logistic and linear regression models were constructed to evaluate differences in dietary intake between the PD and HC groups, as well as within the PD cohort, after controlling for demographic and clinical variables. Correlation of clinically relevant variables was evaluated using Pearson's correlation test. *p* < 0.05 was considered statistically significant. Data analysis was performed using SPSS, version 26 (SPSS Inc, Chicago, Illinois, USA), as described earlier (20).

## Results

### Demographic and Clinical Characteristics

Demographic information pertaining to the cohort studied here has been reported previously (20). In summary, a total of 103 PD patients (56.3% male, mean age 67) and 81 healthy controls (32% male, mean age 62; comprised of 73 spouses, 7 children and 1 sibling) completed the FFQ. Demographic, anthropometric, clinical and nutritional features of the study population are reported in Table 1.

**Table 1:** Cohort demographic and clinical characteristics.

	**Parkinson's disease**	**Healthy control**	**Test statistic**	***p-*value**
Number of Patients, *n* =	103	81		
Mean age (years) [SD, range]^*^	67.1 [12.2, 33–88]	62.4 [15.6, 18–90]	*t* = 2.3 (182)^∧^	**0.023**
Gender - n (%)^*^			χ^2^ *=* 10.7 (1)^∞^	**0.001**
Male	58 (56.3)	26 (32.1)		
Female	45 (43.7)	55 (67.9)		
Marital status - n (%)^*^			χ^2^ = 4.2 (3)^∞^	0.244
Married/de facto	79 (76.7)	69 (85.1)		
Single	10 (9.7)	8 (9.9)		
Widowed	6 (5.8)	1 (1.2)		
Other	8 (7.7)	3 (3.7)		
Ethnicity - n (%)^*^			χ^2^ = 2.3 (3)^∞^	0.506
Caucasian	81 (78.6)	64 (79.0)		
Asian	4 (3.9)	5 (6.2)		
Middle Eastern	7 (6.8)	2 (2.5)		
Other	11 (10.7)	10 (12.3)		
Body mass index [SD]	25.7 [5.2]	26.2 [4.6]	*t* = −0.7 (182)^∧^	0.485
Smoking History - n (%)				
Current smoker	2 (1.9)	3 (3.7)	χ^2^ = 0.6 (1)^∞^	0.457
Prior smoker	38 (36.9)	27 (33.7)	χ^2^ = 0.2 (1)^∞^	0.659
Never smoked	65 (63.1)	53 (66.3)	χ^2^ = 0.2 (1)^∞^	0.659
Type of tobacco (%)			χ^2^ = 2.6 (2)^∞^	0.268
Cigarettes	84.2	96.3		
Cigars	10.5	3.7		
Pipe	5.3	0		
Pack year history, [SD]	13.3 [13.8]	14.4 [14.6]	*t* = −0.3 (63)^∧^	0.758
Caffeine consumption (coffee/tea) (%)	88 (85.4)	74 (91.4)	χ^2^ = 1.5 (1)^∞^	0.219
Number of daily cups, [SD]	2.3 [1.7]	3.1 [1.8]	*t* = 3.0 (182)^∧^	**0.003**
History of diabetes (%)	5 (4.9)	5 (6.2)	χ^2^ = 0.2 (1)^∞^	0.695
Self-Reported HbA1c%, [SD]	6.1 [0.2]	7.3 [1.0]	*t* = −1.9 (6)^∧^	0.095
Biochemical characteristics [SD]^*^				
Erythrocyte sedimentation rate (mm/h)	9.5 [13.4]	9.5 [10.4]	*t* = −0.1 (181)^∧^	0.991
C-Reactive protein (mg/L)	3.9 [10.8]	2.2 [2.5]	*t* = 1.4 (182)^∧^	0.177
Total cholesterol (mmol/L)	4.8 [0.9]	5.2 [1.1]	*t =* −2.5 (182)^∧^	**0.014**
Low density lipoprotein (mmol/L)	2.7 [0.7]	2.9 [0.9]	*t =* −1.5 (178)^∧^	0.132
High density lipoprotein (mmol/L)	1.4 [0.4]	1.6 [0.4]	*t =* −2.2 (181)^∧^	**0.033**
Triglycerides (mmol/L)	1.3 [1.0]	1.5 [0.9]	*t =* −1.2 (182)^∧^	0.239
Random glucose (mmol/L)	5.8 [0.6]	5.9 [0.9]	*t =* −0.8 (182)^∧^	0.438
HbA1c%	5.3 [0.4]	6.0 [5.2]	*t =* −1.2 (182)^∧^	0.217
Albumin (g/L)	38.7 [3.5]	39.8 [3.1]	*t =* −2.3 (182)^∧^	**0.023**
**Dietary variables**				
Vegetarian diet, n (%)	3 (2.9)	2 (2.5)	χ^2^ = 0.1 (1)^∞^	0.865
Energy with dietary fibre (k/J), [SD]	11,131 [5782.6]	10,188 [4799.9]	*t =* 1.2 (181)^∧^	0.241
Energy without dietary fibre (k/J), [SD]	10,778 [5546.6]	9,861 [4624.4]	*t =* 1.2 (181)^∧^	0.235
Protein (g/day) [SD]	118 [79.3]	117 [74.5]	*t =* 0.1 (181)^∧^	0.883
Total fat (g/day) [SD]	102 [49.7]	96 [43.6]	*t =* 0.9 (181)^∧^	0.392
Carbohydrate (g/day) [SD]	279 [161.8]	232 [124.8]	*t =* 2.1 (181)^∧^	**0.034**
Total sugars (g/day) [SD]	153 [86.3]	119 [60.6]	*t =* 3.0 (181)^∧^	**0.003**
Free sugars g/day [SD]	61 [48.0]	41 [23.2]	*t =* 3.5 (181)^∧^	**0.001**
Added sugars g/day [SD]	53 [43.3]	35 [20.4]	*t =* 3.5 (181)^∧^	**0.001**
Fibre (g/day) [SD]	41 [31.2]	38 [22.7]	*t =* 0.7 (181)^∧^	0.475
Moisture (mL/day) [SD]	2,878 [1236.2]	3,044 [1050.6]	*t =* −0.1 (181)^∧^	0.337
Alcohol (g/day) [SD]	9 [12.6]	13 [15.8]	*t =* −2.1 (181)^∧^	**0.038**
Calcium (mg/day) [SD]	1,158 [590.4]	1,129 [593.6]	*t =* 0.3 (181)^∧^	0.739
Iron (mg/day) [SD]	15 [11.0]	14 [8.3]	*t =* 0.4 (181)^∧^	0.677
Magnesium (mg/day) [SD]	478 [270.0]	479 [223.4]	*t =* −0.1 (181)^∧^	0.973
Potassium (mg/day) [SD]	4,965 [3359.4]	4,761 [2551.1]	*t =* 0.5 (181)^∧^	0.652
Sodium (mg/day) [SD]	2,145 [1421.4]	2,075 [1344.2]	*t =* 0.3 (181)^∧^	0.733
Zinc (mg/day) [SD]	14 [8.5]	14 [7.8]	*t =* 0.2 (181)^∧^	0.880
Retinol (ug/day) [SD]	634 [675.0]	533 [545.7]	*t =* 1.1 (181)^∧^	0.281
Beta carotene (ug/day) [SD]	6,703 [7046.0]	6,703 [5805.7]	*t =* 0.1 (181)^∧^	1
Vitamin A (ug/day) [SD]	1,957 [1671.6]	1,867 [1412.3]	*t =* 0.4 (181)^∧^	0.702
Thiamine (mg/day) [SD]	2 [1.0]	2 [0.9]	*t =* 0.1 (181)^∧^	0.955
Riboflavin (mg/day) [SD]	2 [1.2]	2 [1.1]	*t =* 0.1 (181)^∧^	0.663
Vitamin B12 (ug/day) [SD]	7 [4.7]	7 [4.5]	*t =* 0.1 (181)^∧^	0.925
Vitamin C (mg/day) [SD]	158 [147.1]	145 [110.7]	*t =* 0.6 (181)^∧^	0.525
Dietary folate (DFE) (ug/day) [SD]	788 [490.2]	755 [422.2]	*t =* 0.5 (181)^∧^	0.626

∧ *Independent sample t test*;

∞ *Pearson's chi-squared test; df, degrees of freedom; [SD], Standard Deviation*.

* *This data is partially reproduced from Lubomski et al. (20). The bold values indicate clinical significance*.

The mean BMI of the combined cohort was 26.0 (SD 4.90). PD patients were not underweight and their BMI (25.7 [SD 5.2]) did not differ significantly from HC (26.2 [SD 4.6], *p* = 0.485). 5.5% of subjects reported a history of diabetes, with no statistically significant difference observed between the groups for this measure. More PD patients reported chronic pain over the preceding year than HC (72.8 vs. 39.5%, *p* < 0.001). PD patients were also more depressed, as measured by the Beck's Depression Inventory (total score 11.9 [SD 8.8] vs. 5.2 [SD 5.5], *p* < 0.001). PD patients also reported more constipation, as measured by the Cleveland Constipation Score (7.2 [SD 4.7] vs. 3.1 [SD 2.9], *p* < 0.001) and Rome IV Criteria (4.4 [SD 3.5] vs. 1.1 [SD 1.4], *p* < 0.001). Furthermore, PD patients reported more dyspepsia as measured by the Leeds Dyspepsia Questionnaire (score 8.3 [SD 7.7] vs. 4.6 [6.1], *p* = 0.001). Physical activity, assessed by the IPAQ, identified that PD patients undertook considerably less physical activity (1823.6 metabolic-equivalent [MET]-minutes/week [SD 1693.6]) compared to the HC group (2942.4 MET-minutes/week [SD 2620.9], *p* = 0.001). Further clinical characteristics of the PD cohort including the utilisation of standard and device assisted therapies, physical activity and frequency and severity of other non-motor symptoms (NMS) are outlined in Table 2.

**Table 2:** Parkinson's disease clinical characteristics.

Mean age at diagnosis (years) [SD, range]^*^	58.8 [13.6, 24–88]
Mean Parkinson's disease duration (years) [SD, range]^*^	9.2 [6.5, 1–30]
Parkinson's disease phenotype - n (%)^*^	
Tremor dominant	31 (30.1)
Postural instability and gait impairment	21 (20.4)
Akinetic rigid	40 (38.9)
Young onset (<40 years)	11 (10.7)
Late onset (>60 years)	51 (49.5)
Disease complications - n (%)^*^	
Motor fluctuations	60 (58.3)
Dyskinesia	60 (58.3)
Wearing off	84 (81.6)
Impulse control disorder	20 (19.4)
REM sleep behaviour disorder	50 (48.5)
Parkinson's disease therapy - n (%)^*^	
Treatment naïve	5 (4.9)
Oral levodopa	92 (89.3)
Dopamine agonist	36 (35.0)
Monoamine oxidase B inhibitor	19 (18.4)
Anticholinergic	13 (12.6)
Catechol-O-methyl transferase inhibitor	24 (23.3)
Amantadine	13 (12.6)
Levodopa/carbidopa intestinal gel	9 (8.7)
Deep brain stimulation	11 (10.7)
Apomorphine (subcutaneous infusion)	7 (6.8)
Levodopa equivalent daily dose (mg) [SD, range]^*^	834.8 [527.3, 0–2,186]
Mean MDS unified Parkinson's disease rating scale-III (“on” state) [SD, range]^*^	32.9 [17.7, 5–91]
Gastrointestinal symptoms^*^	
Mean cleveland constipation score [SD]	7.2 [4.7]
Mean Rome-IV criteria constipation score [SD]	4.4 [3.5]
Functional constipation as per Rome-IV criteria (%)	78.6
Mean leeds dyspepsia questionnaire (LDQ) score [SD]^*^	8.3 [7.7]
Chronic pain over last 3 months (%)^*^	75 (72.8)
Mean pain score (visual analogue scale) [SD]	4.9 [2.5]
Mean international physical activity questionnaire (IPAQ) score (MET-minutes/week) [SD]^*^	1823.6 [1693.6]
PDQ-39 summary index [SD]	29.2 [17.3]
**Depression characteristics**	
Mean Beck's depression inventory total score [SD]	11.9 [8.8]
Clinically depressed (>13 for Parkinson's disease) - n (%)	40 (38.9)
Mean MDS total non-motor symptoms score (NMSS), [SD]	62.7 [42.9]
Montreal cognitive assessment (MoCA), [SD]	24.4 [4.8]
Mild cognitive impairment (<26/30) - n (%)	50 (48.6)
Parkinson's disease dementia (<21/30) - n (%)	17 (16.5)

*[SD], Standard Deviation*.

* *This data is partially reproduced from Lubomski et al. (20)*.

### Dietary Characteristics

Mean daily energy intake did not differ significantly between PD patients (1130.9 kJ/day [SD 5782.6]) and HC (10188.2 kJ/day [SD 4800.0], *p* = 0.241). When total energy intake was evaluated in terms of gender, the difference between males (11052.4 kJ/day [SD 5486.4]) and females (10435.7 kJ/day [SD 5302.4]) was not statistically different across the whole cohort (*p* = 0.7) or PD cohort alone (males 11350.6 kJ/day [SD 5998.3], females 10847.7 kJ/day [SD 5546.4], *p* = 0.8).

PD patients reported greater total carbohydrate intake compared to HCs (278.8 g/day [SD 161.8] vs. 232.2 g/day [SD 124.8], *p* = 0.034), which was largely attributable to increased daily total sugar intake (153.3 g/day [SD 86.3] vs. 118.7 g/day [SD 60.6], *p* = 0.003; Table 1). Consistently, PD patients consumed more total free sugar (61.2 g/day [SD 48.0] vs. 40.6 g/day [SD 23.2], *p* = 0.001) and total added sugar (52.9 g/day [SD 43.3] vs. 34.7 g/day [SD 20.4], *p* = 0.001) compared to HC. Among people with PD, beverages provide 19.6% of free sugars, compared to about half this among HCs (10.4%). The main contributors to free sugars among both groups were: chocolate, jam/marmalade/honey, cordial, sugar, soft drinks, cake, cold breakfast cereal, and yoghurt.

The total intake of vitamins and other macronutrients did not differ between the groups, and there were no macronutrient or micronutrient differences noted between the genders in both the PD and HC groups. When subjects with diabetes within the PD and combined cohorts were excluded from analysis, PD patients still consumed significantly more carbohydrates, total sugar, added sugar and free sugar than healthy controls (all *p* < 0.05). Excluding PD dementia patients also demonstrated a persistent increased sugar intake compared to HCs.

Logistic regression modelling evaluated the significance of dietary differences between the PD and HC groups. Statistical significance between the two groups persisted after controlling for age, sex, physical activity and constipation (Rome-IV criteria), for the following dietary variables: carbohydrates (Wald χ^2^ = 3.6, df = 3, *p* = 0.044); total sugars (Wald χ^2^ = 3.9, df = 3, *p* = 0.036), free sugars (Wald χ^2^ = 3.5, df = 3, *p* =0.049), added sugars (Wald χ^2^ = 3.6, df = 3, *p* = 0.046) and alcohol (Wald χ^2^ = 4.8, df = 3, *p* = 0.029).

PD patients reported less alcohol consumption compared to the HCs (8.9 g/day [SD 12.6] vs. 13.3 g/day [SD 15.8], *p* = 0.038), with male PD patients consuming more alcohol than female PD patients (12 g/day [SD 14.4] vs. 5 g/day [SD 8.4], *p* = 0.005). Over 90% of participants reported daily caffeine consumption, although PD patients reported lower daily intake (2.3 cups/day [SD 1.7] vs. 3.1 cups/day [SD 1.8], *p* = 0.003). No associations between PD phenotype, standard or advanced therapy use, motor severity (assessed by the MDS-UPDRS-III score), or any of the measured dietary parameters were identified.

**Figure 1:**
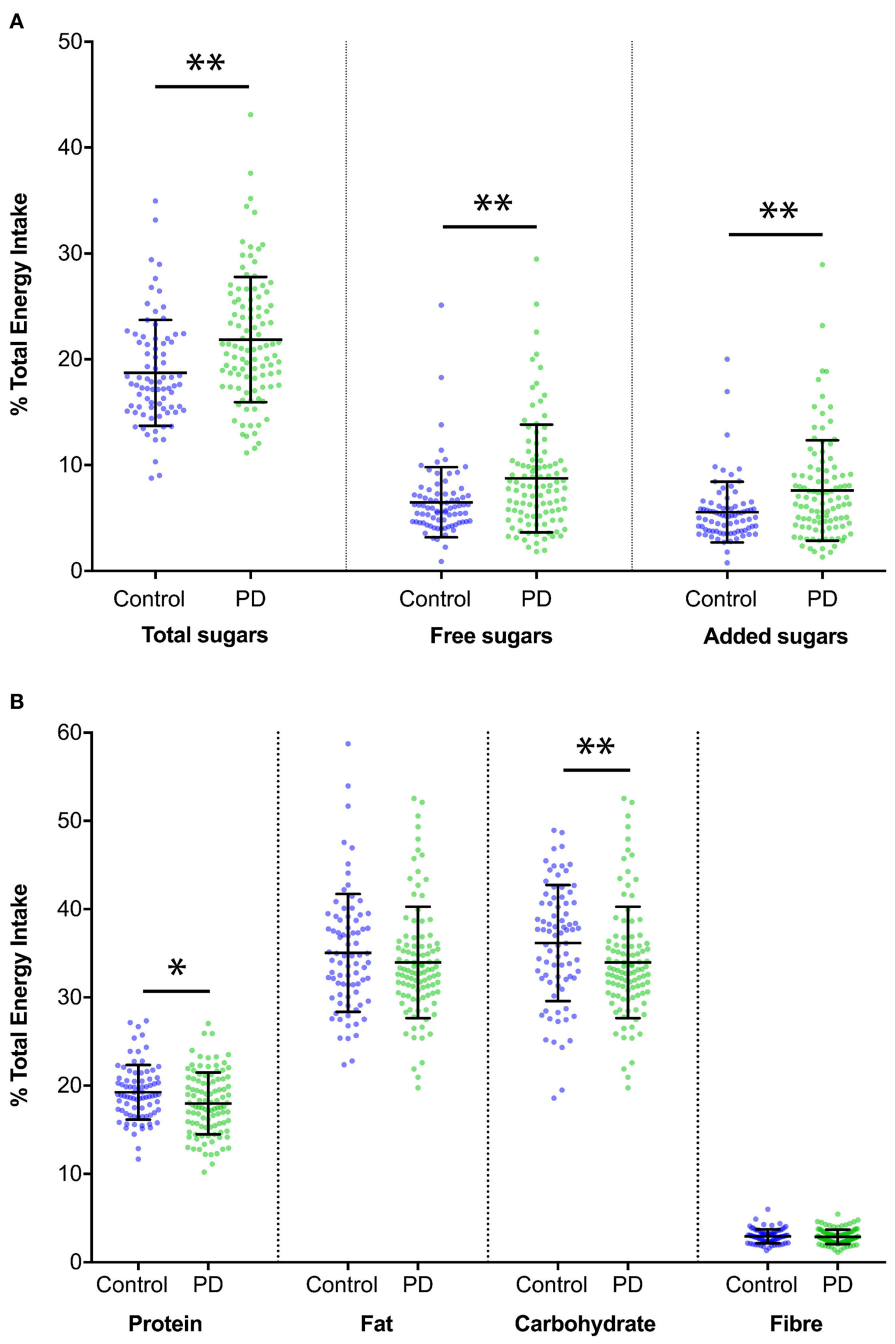
Comparison of macronutrient intake as a percentage of total energy intake. **(A)** Comparison of sugar intake expressed as a percentage of total energy intake between Parkinson's disease and healthy control groups (Mean ± [SD]). Patients with Parkinson's disease consumed a greater amount of total sugars (percentage of total energy intake; 21.9% [5.9] in Parkinson's disease patients vs. 18.7% [5.0] in healthy controls, *p* < 0.001). Similar findings were also noted for free sugars (8.8% [5.1] vs. 6.5% [3.3], *p* = 0.001) and added sugars (7.6% [4.7] vs. 5.6% [2.9], *p* = 0.001). Statistically significant comparisons of *p* ≤ 0.001 are indicated with**. **(B)** Comparison of protein, fat, carbohydrate and fibre intake for Parkinson's disease and Healthy control groups, expressed as a percentage of total energy intake (Mean ± [SD]). Statistically significant comparisons of *p* ≤ 0.05 are indicated with*.

**Figure 2:**
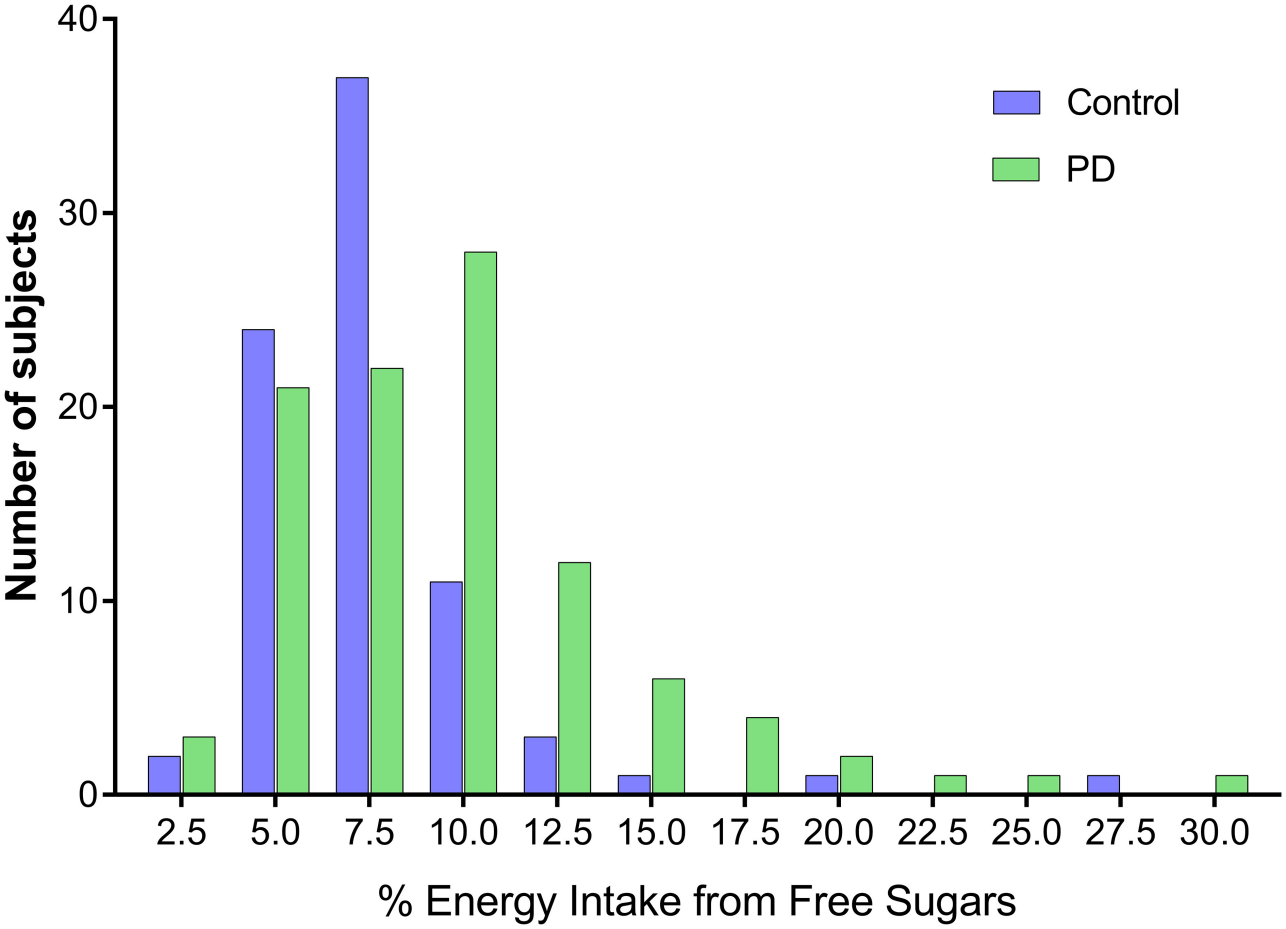
Comparison of relative energy intake from free sugars between Parkinson's disease patients and healthy controls. Parkinson's disease patients consumed more free sugars as a proportion of energy intake compared to healthy controls.

Evaluating macronutrient intake calculated as a percentage of total energy intake, PD patients consumed less protein than HCs (18.0% [SD 3.5] vs. 19.2% [SD 3.1], *p* = 0.011), as well as more carbohydrates (40% [SD 6.1] vs. 36.2% [SD 6.6], *p* < 0.001) and more added sugar (7.6% [SD 4.7] vs. 5.6% [SD 2.9], *p* = 0.001). PD patients also consumed more total sugars (21.9% [SD 5.9] vs. 18.7% [SD 5.0], *p* < 0.001) and more free sugars (8.8% [SD 5.1] vs. 6.5% [SD 3.3], *p* = 0.001), when expressed as a percentage of total energy intake (Figure 1 and Table 3). Moreover, the percentage total energy consumption of free sugars showed that 28.2% of PD patients compared with 7.5% of HC had >10% of energy intake attributed to free sugars (Figure 2). The assessment of micronutrients, expressed per 1,000 kJ energy intake, identified that PD patients consumed less magnesium, potassium and zinc than HC (Table 3). Ten participants (6 PD and 4 HC) reported a very high energy consumption. Reanalysis of the combined cohort when excluding these individuals did not alter the significance of the macro- and micronutrient findings between the PD and HC groups, both in terms of total intake and percentage of total energy intake. When controlling for participant age and sex, using linear regression analysis with Bonferroni correction for multiple testing (new α threshold; *p* < 0.016), all statistically significant comparisons remained, aside from potassium (Table 3).

**Table 3:** Intake of macronutrients expressed as percentage of energy intake and intake of micronutrients expressed per 1,000 kJ intake.

	**Parkinson's disease**	**Healthy control**	**Test statistic**	***p-*value**
Number of patients (*n* =)	103	81		
**Dietary variables**				
Protein % [SD]	18.0 [3.5]	19.2 [3.1]	*t =* −2.6 (181)^∧^	0.011^*^
Total fat % [SD]	34.0 [6.3]	35.0 [6.7]	*t =* −1.1 (181)^∧^	0.262
Carbohydrate % [SD]	40.0 [6.1]	36.2 [6.6]	*t =* 4.0 (181)^∧^	<0.001^*^
Total Sugars % [SD]	21.9 [5.9]	18.7 [5.0]	*t =* 3.8 (181)^∧^	<0.001^*^
Free sugars % [SD]	8.8 [5.1]	6.5 [3.3]	*t =* 3.4 (181)^∧^	0.001^*^
Added sugars % [SD]	7.6 [4.7]	5.6 [2.9]	*t =* 3.4 (181)^∧^	0.001^*^
Fibre % [SD]	2.9 [0.8]	2.9 [0.8]	*t =* −0.5 (181)^∧^	0.606
Alcohol % [SD]	2.5 [3.6]	4.1 [4.7]	*t* = −2.6 (181)^∧^	0.010^*^
Calcium (mg/day per 1,000 kJ) [SD]	106.8 [33.5]	111.3 [30.1]	*t =* 1.0 (181)^∧^	0.343
Iron (mg/day per 1,000 kJ) [SD]	1.3 [0.3]	1.3 [0.2]	*t =* −1.5 (181)^∧^	0.141
Magnesium (mg/day per 1,000 kJ) [SD]	43.0 [8.0]	47.0 [6.5]	*t =* −3.6 (181)^∧^	<0.001^*^
Potassium (mg/day per 1,000 kJ) [SD]	440.1 [84.1]	465.4 [67.1]	*t =* −2.2 (181)^∧^	**0.029**
Sodium (mg/day per 1,000 kJ) [SD]	192.3 [51.3]	201.7 [49.3]	*t =* −1.3 (181)^∧^	0.213
Zinc (mg/day per 1,000 kJ) [SD]	1.2 [0.2]	1.3 [0.2]	*t =* −3.0 (181)^∧^	0.003^*^
Retinol (ug/day per 1,000 kJ) [SD]	61.4 [83.1]	53.4 [57.1]	*t =* 0.7 (181)^∧^	0.490
Beta carotene (ug/day per 1,000 kJ) [SD]	585.9 [326.6]	648.3 [434.7]	*t =* −1.1 (181)^∧^	0.269
Vitamin A (ug/day per 1,000 kJ) [SD]	177.0 [100.7]	183.0 [100.9]	*t =* −0.4 (181)^∧^	0.690
Thiamine (mg/day per 1,000 kJ) [SD]	0.2 [0.0]	0.2 [0.0]	*t =* −1.9 (181)^∧^	0.060
Riboflavin (mg/day per 1,000 kJ) [SD]	0.2 [0.1]	0.2 [0.1]	*t =* −0.9 (181)^∧^	0.372
Vitamin B12 (ug/day per 1,000 kJ) [SD]	0.6 [0.3]	0.7 [0.2]	*t =* −0.9 (181)^∧^	0.365
Vitamin C (mg/day per 1,000 kJ) [SD]	13.7 [8.0]	14.2 [7.8]	*t =* −0.5 (181)^∧^	0.643
Dietary folate (DFE) (ug/day per 1,000 kJ) [SD]	70.5 [17.0]	73.6 [16.0]	*t =* −1.3 (181)^∧^	0.203

∧ *Independent sample t test; df, degrees of freedom; [SD], Standard Deviation*.

* *Indicates dietary variables that remain statistically significant after Bonferroni correction (p < 0.016) of multiple testing with linear regression modelling when controlling for participant age and sex, as potential confounders. The bold values indicate clinical significance*.

### Biochemical Characteristics

Biochemical analysis showed that PD patients had lower total cholesterol levels (4.8 mmol/L [SD 0.9] vs. 5.2 mmol/L [SD 1.1], *p* = 0.014), lower high density lipoprotein (HDL) levels (1.4 mmol/L [SD 0.4] vs. 1.6 mmol/L [SD 0.4], *p* = 0.033), and lower albumin levels (38.7 mmol/L [SD 3.5] vs. 39.8 mmol/L [SD 3.1], *p* = 0.023), although all measures were still within normal physiological ranges. A full biochemical description is provided in Table 1.

### Dietary Intake of the Parkinson's Disease Cohort

#### Impulse Control Disorders

Mean energy intake was significantly greater for PD patients who reported an impulse control disorder compared to those without (13544.3 kJ/day [SD 8357.8] vs. 10549.3 kJ/day [SD 4862.8], *p* = 0.037), after adjusting for age, sex and PD duration (β = −0.203, *r*^2^ = 0.073, *p* = 0.041). This was mainly attributable to increased consumption of carbohydrates (353.9 g/day [SD 246.3] vs. 260.8 [SD 129.7] g/day, *p* = 0.020), increased total sugar intake (199.5 g/day [SD 120.2] vs. 142.2 g/day [SD 72.7], *p* = 0.007) and increased consumption of total fibre (57.3 g/day [SD 43.0] vs. 37.2 g/day [SD 26.7], *p* = 0.009) by PD patients with an impulse control disorder. Linear regression modelling validated that the increased carbohydrate and total sugar consumption in impulse control disorder patients persisted after controlling for patient age, sex and PD duration (β = −0.229, *r*^2^ = 0.084, *p* = 0.021, and β = −0.263, *r*^2^ = 0.101, *p* = 0.008, respectively). PD patients with an impulse control disorder also consumed greater amounts of a variety of vitamins and minerals, as outlined in Supplementary Table 1. When micronutrient intake was assessed per 1,000 kJ energy intake, PD patients with an impulse control disorder compared to PD patients without an impulse control disorder consumed more potassium (483.7 mg/day [SD 76.7] vs. 429.6 mg/day [SD 82.8], *p* = 0.009), more beta carotene (723.4 ug/day [SD 348.1] vs. 522.7 ug/day [SD 314.5], *p* = 0.035) and more vitamin C (17.3 mg/day [SD 11.2] vs. 12.8 mg/day [SD 6.7], *p* = 0.022).

#### Depression

PD patients who were depressed (BDI >13) (46), consumed more added sugars compared to those who were not depressed (63.7 g/day [SD 43.6] vs. 46.1 g/day [SD 42.0], *p* = 0.043), after controlling for patient age, sex and PD duration (β = −0.192, *r*^2^ = 0.062, *p* = 0.040). Interestingly, depressed PD patients consumed less alcohol than those who did not report depression (5.6 g/day [SD 9.1] vs. 11.0 g/day [SD 14.1], *p* = 0.034), after controlling for patient age, sex and PD duration (β = 0.195, *r*^2^ = 0.061, *p* = 0.044).

#### Cognition

Those PD patients meeting the criteria for PD dementia (MoCA <21/30) and loss of one or more instrumental activities of daily living (47), consumed significantly more total sugar per day (195.1 g/day [SD 67.8] vs. 145.1 g/day [SD 87.5], *p* = 0.028), total free sugar (87.5 g/day [SD 53.5] vs. 56 g/day [SD 45.4], *p* = 0.013), and total added sugars (77.3 g/day [SD 51.1] vs. 48.1 g/day [SD 40.1], *p* = 0.010) compared to PD patients without dementia, after controlling for patient age, sex and PD duration (β = −0.207, *r*^2^ = 0.111, *p* = 0.033; β = −0.213, *r*^2^ = 0.111, *p* = 0.031; β = −0.225, *r*^2^ = 0.117, *p* = 0.023, respectively).

#### Chronic Pain and Other Clinical Features

PD patients with chronic pain consumed more total sugar than PD patients without chronic pain (164.0 g/day [SD 92.2] vs. 124.6 g/day [SD 60.8], *p* = 0.039; controlling for age, sex and PD duration, β = −0.202, *r*^2^ = 0.087, *p* = 0.040). Patients with REM sleep behaviour disorder (RBD) reported significantly more total sugar consumption per day compared with PD patients without RBD (174.2 g/day [SD 96.6] vs. 133.6 g/day [SD 70.8], *p* = 0.016; β = −0.208, *r*^2^ = 0.087, *p* = 0.020) after controlling for patient age, sex and PD duration. PD patients with RBD consumed more total free sugars (77.6 g/day [SD 59.5] vs. 45.8 g/day [SD 26.0], *p* = 0.001) and total added sugars (67.0 g/day [SD 54.3] vs. 39.6 g/day [SD 22.9], *p* = 0.001) compared to those without RBD, after controlling for patient age, sex and PD duration (β = −0.320, *r*^2^ = 0.132, *p* = 0.001 and β = −0.306, *r*^2^ = 0.125, *p* = 0.002 respectively). PD patients with motor fluctuations consumed less alcohol than those without motor fluctuations (6.3 g/day [SD 9.7] vs 12.6 g/day [SD 15.2], *p* = 0.013), after controlling for patient age, sex and PD duration (β = 0.161, *r*^2^ = 0.379, *p* = 0.049), possibly due to alcohol further exacerbating their brittle PD motor features. When adjusted for energy intake per 1,000 kJ, PD patients with dyskinesia consumed more beta carotene (8060.4 ug/day [SD 8759.2]) compared to those without dyskinesia (4809.3 ug/day [SD 2547.1], *p* = 0.024).

#### Dietary and Clinical Correlations

PD patient age was negatively correlated with the amount of daily protein intake (*r* = −0.277, *p* = 0.005). Furthermore, increasing PD duration was associated with a lower albumin level (*r* = −0.208, *p* = 0.004). Increased alcohol consumption was associated with increased age at diagnosis (*r* = 0.201, *p* = 0.042) and older age at commencing treatment (*r* = 0.200, *p* = 0.026). Higher PDQ-39 SI scores (suggesting poorer QoL) were associated with lower total alcohol consumption (*r* = −0.31, *p* = 0.001) and higher total free sugar consumption (*r* = 0.248, *p* = 0.012). That is, PD individuals with a worse QoL consumed less alcohol, but ingested more sugar. Increased constipation severity was associated with increased free and added sugar consumption (*r* = 0.211, *p* = 0.032; *r* = 0.201, *p* = 0.042), respectively. Increased total sugar consumption was associated with greater daily levodopa dose (LED) requirement (*r* = 0.272, *p* = 0.005) and greater burden of non-motor symptoms as measured by the NMSS (*r* = 0.213, *p* = 0.031). The above associations of excess sugar consumption in patients with more severe constipation, higher LED and worse NMSS scores can be partially explained by features suggestive of advancing PD severity. Higher NMSS total scores were also associated with higher total fat intake (*r* = 0.292, *p* = 0.003), increased protein consumption (*r* = 0.232, *p* = 0.018) and overall higher mean energy intake (*r* = 0.257, *p* = 0.009), suggesting that individuals who were more burdened by NMS required an increased food intake that was higher in protein and fat. Lastly, the effects of free sugar intake were also associated with gastrointestinal dysfunction in PD, with individuals who consumed more free sugars also reporting worse constipation, noted by the Rome-IV criteria (*r* = 0.195, *p* = 0.049), and Cleveland Constipation Score (*r* = 0.211, *p* = 0.032), as well as worse upper gastrointestinal dysfunction, indicated by a higher Leeds Dyspepsia Questionnaire score (*r* = 0.202, *p* = 0.040).

## Discussion

The results of this study provide novel and clinically important insights into the dietary habits of Australian PD patients. Consistent with other reports (7, 15, 16), PD patients consumed greater amounts of carbohydrates, which was largely attributable to increased daily sugar intake. However, in contrast, Barichella et al. (7) found increased consumption of many other macro and micronutrients by PD patients, rather than carbohydrates and sugars alone, as in our cohort. There are several reasons why PD patients may consume more sugar. It has been suggested that carbohydrates and sweets, through insulin, may increase brain dopamine as somewhat of a compensatory mechanism for disease-related dopamine loss (14, 15). A variety of mechanisms contribute to altered eating behaviour in PD, such as alterations in hypothalamic regulation, energy expenditure and dopaminergic signalling (48). Food reward alterations seem to be present in PD, and these may influence eating behaviours (49). In addition, non-motor complications affecting taste and olfaction, cognition, mood and reward may impair food perception, eating behaviours and motivation toward food consumption (49). Perhaps seeking more sugary foods is related to a decrease in taste function in PD patients. As demonstrated by Cecchini et al. (50), PD patients have reduced olfactory function and taste performance compared with controls. A chemosensory interaction has been proposed, where olfactory loss leads to a decrease in taste function (51).

Concerningly, we have shown a generally unhealthy diet in many PD patients in our study. In 2015 the World Health Organisation (WHO) issued a recommendation that both adults and children reduce their intake of free sugars to <10% of total dietary energy to help reduce the non-communicable disease burden from unhealthy weight gain and dental caries (33). Notably, in this study, 28.2% of PD patients compared to 7.5% of HCs had >10% energy intake attributed to free sugars (Figure 2), which is outside of the recommended WHO guidelines for healthy eating (52). Reassuringly, the PD cohort sampled did not have greater prevalence of diabetes, or a higher HbA1c than HCs. However, these measures should be monitored carefully throughout. Further evidence of an unhealthy diet is demonstrated by our PD patients consuming lower levels of certain micronutrients (when expressed per 1,000 kJ energy intake), despite high levels of sugar consumption. This is consistent with previous research, which has shown that intake of added sugar greater than the recommended level of 10% is associated with lower micronutrient intakes, indicating micronutrient dilution (53). The macronutrient distribution in this study cohort is similar to those reported for an Australian population, as per The Australian Health Survey 2011–13 (54). Poor diet in PD has been shown in a number of previous studies, for example in a study by van Steijn et al. (55) of Dutch elderly PD patients, 22.5% had unfavourable nutritional status. Patients in this study consumed less protein than HCs (when expressed as a percentage of energy intake), which is consistent with the finding here of slightly lower serum albumin levels, suggesting poorer nutritional status. Lower protein consumption by PD patients has not consistently been found (7, 16), despite a low protein or protein redistribution diet being recommended for PD patients with motor fluctuations (56, 57). It is known that the absorption of one of the most commonly used oral medications for PD, Levodopa, is impaired by simultaneous protein ingestion, and thus may be a potential reason why more PD patients eat less protein routinely (57).

In this study, patients who reported impulse control disorders consumed more sugar. Eating disorders are common in PD, and 21.6% of PD patients experience episodes of out-of-control eating with a large quantity of food in short time (58). The existence of a food addiction profile has been described in PD patients, and more specifically compulsive eating symptomatology (58). It is possible that the PD patients in our cohort binge eat, although this was not evident from the measures used and is not a feature considered in the impulse control disorder questionnaire. Dietary intake and compulsive eating in PD patients with impulse control disorders warrants further investigation.

Furthermore, increased sugar consumption was associated with chronic pain and depression. Depression is prevalent in PD, with 38.9% of our PD cohort reporting depression, which is almost double the proportion of depressed HCs (59). More PD patients in our study reported depression and chronic pain than healthy controls, which may contribute to the increased sugar consumption of these patients compared to controls. Depression may alter appetite, food intake and weight regulation. Serotonin plays a role in eating behaviour, and as discussed in a review by Kistner et al. (48), neurodegeneration of the serotonergic system, with low levels of serotonin in PD, may explain the pronounced preference for sweet foods. The association between sugar consumption and indicators of disease severity (e.g., greater LED, more non-motor symptoms) may suggest the possibility of comfort eating behaviour. Furthermore, cognition plays an important role in eating behaviour (49) meaning our results may have been influenced by the inclusion of patients with cognitive impairment and dementia, whereas many other studies exclude patients with MMSE <24. However, when subjects with dementia are excluded from analysis, the finding of increased sugar consumption by PD patients compared with HCs persisted.

PD patients in our cohort consumed less alcohol than healthy controls, which is consistent with previous findings (7, 13, 16). A possible explanation for this is that PD patients may be replacing alcoholic drinks with sugar sweetened beverages, as in previous research where higher added sugar intake has been associated with lower alcohol intake (53). Another proposed explanation for lower alcohol consumption by PD patients is that they may fear potential interactions between alcohol and medications (14). PD patients who reported motor fluctuations were found to consume less alcohol, perhaps suggesting a reluctance to consume alcohol for fear of worsening tremor or other motor features. Likewise, PD patients who were depressed potentially consumed less alcohol due to suspected medication interactions or perceptions of alcohol worsening their mood or PD management.

The mean BMI in our cohort was 26 and suggests that participants were overweight, consistent with epidemiological data in Australia (50). However, BMI did not differ between PD patients and HC. This is contrary to prior findings of lower weight and BMI in PD patients (3, 4, 6, 7), but consistent with other studies showing no difference in BMI (16). A possible explanation for this may be the relatively affluent socioeconomic standing of our cohort and the fact that our HC group were spouses of the PD patients.

The findings of this study are limited by its relatively small cohort size and cross-sectional design. Dietary habits may change over time, are typically influenced by seasonal availability of certain foods and are influenced by multiple disease factors. No significant relationship was seen between PD duration and sugar consumption. Over the course of the disease, nutritional requirements may change, body weight may fluctuate, with changes in both energy expenditure and food intake (10). Longitudinal studies, with larger sample size, are needed to further evaluate these dietary trends. Another limitation of this study is the potential for selection bias, with the population drawn from a single specialist PD centre, and in an area of relatively high socioeconomic status in metropolitan Sydney, Australia. Whereas, previous Australian studies have shown PD patients from regional areas to be comparably older with an older age of diagnosis and comparatively lower socioeconomic status (60, 61). Furthermore, the FFQ is subject to recall bias, particularly the reliance on long-term memory and errors in estimating frequencies and serving sizes. Memory recall may be unreliable in patients with cognitive impairment. Furthermore, the FFQ has also been shown to have a tendency to overestimate total carbohydrate and sugar (26), which may be relevant given our findings, although partially controlled for as both PD and HC cohorts completed the same FFQ. Mean fibre intake in our cohort is generally high and may be overestimated by the FFQ assessment. However, even when subjects with very high energy consumption are excluded from analysis, the significant findings observed in this study persist. Additionally, the comparability of these results to other studies is limited by the different dietary assessment tools utilised. Dietary habits vary significantly depending on ethnicity (22), limiting the generalizability of our findings. However, this also highlights the importance of this research, being the only dietary data to our knowledge for an Australian population of PD patients.

Important clinical correlations were identified in this study, such as increased sugar consumption being associated with an increase in non-motor symptoms, poorer QoL, increased constipation severity and greater levodopa requirements. Adherence to a healthy diet has recently been shown to reduce the occurrence of non-motor symptoms that predate PD diagnosis (24). It therefore remains to be determined if a reduction in dietary intake of added sugar can consequently reduce disease complications and non-motor features. Further research on dietary variations and their associations with clinical PD features and complications is warranted. Additionally, the high consumption of added sugar in our cohort highlights the need to carefully monitor PD patients for the development of diabetes.

## Conclusion

Evaluating the dietary habits of an Australian PD cohort has provided valuable insights into important clinical associations between diet and disease characteristics. Thorough management of patient nutrition should be considered integral to patient care, as nutrition associates with many disease complications. We encourage clinicians to promote healthy eating as part of routine clinical care. The WHO strongly recommend reducing free sugar intake to <10% to provide health benefits (52), and PD patients are at particular risk of the consequences of excess sugar consumption shown here. PD patients with impulse control disorders, RBD, depression, cognitive impairment, chronic pain and motor fluctuations are at risk of specific variations in nutritional intake, in particular excess consumption of added sugars. PD patients would benefit from dietitian input as part of routine clinical management.

## Data Availability Statement

The raw data supporting the conclusions of this article will be made available by the authors, without undue reservation.

## Ethics Statement

The studies involving human participants were reviewed and approved by the Northern Sydney Local Health District Human Research Ethics Committee and the North Shore Private Hospital Ethics Committee, HREC/18/HAWKE/109, NSPHEC 2018-LNR-009, respectively. The patients/participants provided their written informed consent to participate in this study.

## Author Contributions

NP and VF: study design, analysed data, drafted, and reviewed the manuscript. ML: study design, reviewed patients, collected and analysed data, drafted, and reviewed the manuscript. RD and CS: study design, supervision, drafted, and reviewed the manuscript. All authors contributed to the article and approved the submitted version.

## Conflict of Interest

The authors declare that the research was conducted in the absence of any commercial or financial relationships that could be construed as a potential conflict of interest. The reviewer JW declared a shared affiliation with the authors, VF to the handling editor at time of review.
