# Supplementary material for: Increased Added Sugar Consumption Is Common in Parkinson's Disease
Source: Front Nutr. 2021 May 7;8:628845. doi: 10.3389/fnut.2021.628845 (PMC8138322; doi:10.3389/fnut.2021.628845)
Supplement: Supplementary file 1 [file Table_1.docx]

**Supplementary Table 1: Parkinson’s Disease Dietary and Clinical Associations**

|  |  |  |  | **Test Statistic** |  | **p value** |
| --- | --- | --- | --- | --- | --- | --- |
| **Impulse Control Disorder** |  | **Present (n=20)** | **Absent (n=83)** |  |  |  |
| Energy with dietary fibre (kJ/day) [SD] |  | 13544 [8357.8] | 10549 [4862.8] | *t*=2.1 (101)^ |  | **0.037** |
| Energy without dietary fibre (kJ/day) [SD] |  | 13054 [8016.1] | 10229 [4670.2] | *t*=2.1 (101)^ |  | **0.040** |
| Moisture (mL/day) [SD] |  | 3534 [1765.6] | 2720 [1023.8] | *t*=2.7 (101)^ |  | **0.008** |
| Fibre (g/day) [SD] |  | 57 [43.0] | 37 [26.7] | *t*= 2.7 (101)^ |  | **0.009** |
| Total sugars (g/day) [SD] |  | 200 [120.2] | 142 [72.7] | *t*= 2.7 (101)^ |  | **0.007** |
| Carbohydrates (g/day) [SD] |  | 354 [246.3] | 261 [129.7] | *t*=2.3 (101)^ |  | **0.020** |
| Magnesium (mg/day) [SD] |  | 612 [367.4] | 445 [232.2] | *t*=2.6 (101)^ |  | **0.012** |
| Potassium (mg/day) [SD] |  | 6645 [4441.5] | 4560 [2934.8] | *t*=2.6 (101)^ |  | **0.012** |
| Zinc (mg/day) [SD] |  | 17 [12.7] | 13 [7.0] | *t*= 2.1 (101)^ |  | **0.037** |
| Beta carotene (ug/day) [SD] |  | 10134 [8672.3] | 5876 [6384.8] | *t*=2.5 (101)^ |  | **0.015** |
| Vitamin A (ug/day) [SD] |  | 2714 [2086.4] | 1774 [1514.5] | *t*=2.3 (101)^ |  | **0.023** |
| Thiamine (mg/day) [SD] |  | 2 [1.6] | 2 [0.8] | *t*= 2.3 (101)^ |  | **0.026** |
| Riboflavin (mg/day) [SD] |  | 3 [1.8] | 2 [0.9] | *t*= 2.3 (101)^ |  | **0.024** |
| Dietary folate (ug/day) [SD]  Vitamin C (mg/day) [SD] |  | 1057 [775.4]  251 [244.7] | 723 [371.0]  135 [102.0] | *t*=2.8 (101)^  *t*= 3.3 (101)^ |  | **0.006**  **0.001** |
| **Depression** |  | **Present (n=20)** | **Absent (n=63)** |  |  |  |
| Alcohol (g/day) [SD] |  | 6 [9.1] | 11 [14.1] | *t*=-2.1 (101)^ |  | **0.034** |
| Added sugars (g/day) [SD] |  | 64 [43.6] | 46 [42.0] | *t*=2.1 (101)^ |  | **0.043** |
| **Chronic pain** |  | **Present (n=75)** | **Absent (n=28)** |  |  |  |
| Total sugars (g/day) [SD] |  | 164 [92.2] | 125 [60.8] | *t*=2.1 (101)^ |  | **0.039** |
| **Dyskinesia** |  | **Present (n=60)** | **Absent (n=43)** |  |  |  |
| Beta carotene (ug/day) [SD] |  | 8060 [8759.2] | 4810 [2547.1] | *t*=2.4 (101)^ |  | **0.020** |
| Vitamin A (ug/day) [SD] |  | 2289 [2053.2] | 1493 [697.4] | *t*=2.4 (101)^ |  | **0.016** |
| Dietary folate (ug/day) [SD] |  | 870 [586.2] | 674 [279.1] | *t*=2 (101)^ |  | **0.045** |
| Vitamin C (mg/day) [SD] |  | 183 [170.8] | 123 [97.1] | *t*=2.1 (101)^ |  | **0.041** |
| **Motor fluctuations** |  | **Present (n=60)** | **Absent (n=43)** |  |  |  |
| Alcohol (g/day) [SD] |  | 6 [9.7] | 13 [15.2] | *t*=-2.5 (101)^ |  | **0.013** |
| **REM Sleep Behaviour Disorder (RBD)** |  | **Present (n=50)** | **Absent (n=53)** |  |  |  |
| Total sugars (g/day) [SD] |  | 174 [96.6] | 134 [70.8] | *t*=2.4 (101)^ |  | **0.016** |
| Free sugars (g/day) [SD] |  | 78 [59.5] | 46 [26.0] | *t*=3.5 (101)^ |  | **0.001** |
| Added sugars (g/day) [SD] |  | 67 [54.3] | 40[22.9] | *t*=3.4 (101)^ |  | **0.001** |
| **Dementia** |  | **Present (n=17)** | **Absent (n=86)** |  |  |  |
| Total sugars (g/day) [SD] |  | 195 [SD 67.8] | 145 [87.5] | *t*=2.2 (101)^ |  | **0.028** |
| Free sugars (g/day) [SD]  Added sugars (g/day) [SD] |  | 88 [53.5]  77 [51.1] | 56 [45.4]  48 [40.1] | *t*=2.5 (101)^  *t=*2.6 (101)^ |  | **0.013**  **0.010** |

^(Independent sample *t* test), [SD] = Standard deviation, (df) = degrees of freedom
